# Managing innovation: a qualitative study on the implementation of telehealth services in rural emergency departments

**DOI:** 10.1186/s12913-022-08271-0

**Published:** 2022-07-02

**Authors:** Mochamad Muska Nataliansyah, Kimberly A. S. Merchant, James A. Croker, Xi Zhu, Nicholas M. Mohr, James P. Marcin, Hicham Rahmouni, Marcia M. Ward

**Affiliations:** 1 Department of Surgery, Division of Surgical Oncology, Collaborative for Healthcare Delivery Science, Medical College of Wisconsin, 8701 Watertown Plank Road, Milwaukee, WI 53005 USA; 2 Department of Health Management and Policy, College of Public Health, University of Iowa, Iowa City, USA; 3 Cardiovascular Research Institute, University of California School of Medicine, San Francisco, CA USA; 4 Department of Health Policy and Management, Fielding School of Public Health, University of California, Los Angeles, CA USA; 5 Department of Emergency Medicine, Carver College of Medicine, University of Iowa, Iowa City, USA; 6 University of California Davis School of Medicine, Sacramento, CA USA; 7 Richard G. Lugar Center for Rural Health, Union Health, Terre Haute, IN USA

**Keywords:** Telehealth, Rural hospitals, Implementation, Adoption, Innovation

## Abstract

**Background:**

Telehealth studies have highlighted the positive benefits of having the service in rural areas. However, there is evidence of limited adoption and utilization. Our objective was to evaluate this gap by exploring U.S. healthcare systems’ experience in implementing telehealth services in rural hospital emergency departments (TeleED) and by analyzing factors influencing its implementation and sustainability.

**Methods:**

We conducted semi-structured interviews with 18 key informants from six U.S. healthcare systems (hub sites) that provided TeleED services to 65 rural emergency departments (spoke sites). All used synchronous high-definition video to provide the service. We applied an inductive qualitative analysis approach to identify relevant quotes and themes related to TeleED service uptake facilitators and barriers.

**Results:**

We identified three stages of implementation: 1) the start-up stage; 2) the utilization stage; and 3) the sustainment stage. At each stage, we identified emerging factors that can facilitate or impede the process. We categorized these factors into eight domains: 1) strategies; 2) capability; 3) relationships; 4) financials; 5) protocols; 6) environment; 7) service characteristics; and 8) accountability.

**Conclusions:**

The implementation of healthcare innovation can be influenced by multiple factors. Our study contributes to the field by highlighting key factors and domains that play roles in specific stages of telehealth operation in rural hospitals. By appreciating and responding to these domains, healthcare systems may achieve more predictable and favorable implementation outcomes. Moreover, we recommend strategies to motivate the diffusion of promising innovations such as telehealth.

## Background

Telehealth is a promising healthcare innovation that can improve access to medical services and address rural health challenges [1, 2]. Despite its potential, healthcare providers have not widely adopted and utilized telehealth in rural areas [3–5]. This adoption gap highlights the importance of characterizing and explaining telehealth implementation approaches to better understand why some telehealth operations succeed while others are not sustained [6].

Prior research on factors that influence telehealth adoption focused on large healthcare systems and general healthcare settings [7–9]. However, these studies have not explored important specialized settings, clinical applications, and factors influencing its implementation [10–12]. This study aims to explore healthcare systems’ experience in implementing telehealth services in rural hospital emergency departments (TeleED) and analyze the facilitators and barriers influencing its implementation and sustainability. Using data collected from six U.S. healthcare systems that provided TeleED services, we aim to identify themes and provide valuable insights regarding recommended approaches to telehealth service delivery in rural hospitals. Future adopters can consider key elements outlined by this study to achieve favorable outcomes from their telehealth implementation efforts.

## Methods

### Sample

The six U.S. healthcare systems that participated in this study were the Evidence-Based Tele-Emergency Network Grant Program (EB TNGP) recipients. This grant program from the Health Resources and Services Administration (HRSA) was designed to support implementation and evaluation of telehealth networks’ delivery of TeleED consultation services to rural hospitals lacking local emergency medicine specialists [13].

The six healthcare system grant recipients all served as the hub for their TeleED services. Across the six, they provided TeleED services to 65 rural EDs (spoke sites) in 11 U.S. states and all used synchronous high-definition video to do so. Among the six grant recipients, three provided general TeleED services, while the remaining three provided specialized TeleED services (i.e., stroke, behavioral health, pediatric critical care). The six healthcare systems varied considerably in how their services were structured and delivered for individual encounters.

### Data collection

Experienced qualitative researchers at a data coordinating center together conducted semi-structured interviews by telephone. One interviewer directs the data coordinating center and the other is senior staff. Both have years of experience conducting telehealth research and qualitative methods. The interviewees were 18 key informants (principal investigators or project directors) from within each participating TeleED hub. Their roles were evaluating the TeleED service operations throughout the period of implementation. The study was focused on the hub to gather insights from the interviewees about the entire implementation process. This unique perspective provides a meta-synthesis in that the interviewees shared their hub’s organizational perspective on barriers and facilitators to implementing TeleED services across multiple rural hospitals. We conducted 11 interviews between 2016 and 2018 to capture insights from different TeleED service delivery phases. Specifically, four interviews were conducted in 2016, three interviews in 2017, and four interviews in 2018. Overall, the stages of implementation across all hubs followed the same trajectory, in accordance with the funding period. Even though each hub might have different characteristics, the similar trajectory allowed the researchers to conduct data collection on the designated time frame (i.e., the beginning, the middle, and the end of the funding period). We recorded the contexts’ variation across the hubs while focused on gathering shared themes derived from the various implementation experiences. Some of the interviewees reflected on the experiences they shared in previous interviews. We addressed these occurrences by flagging them appropriately during the analysis process.

An interview guide was used as an outline to discuss and collect detailed information on TeleED operations from the perspective of TeleED hub leadership. Interviewers explored specific topics such as the characteristics of the hub and spoke facilities, facilitators and barriers in implementing and delivering TeleED services, the business model, and the type of services provided. Also, TeleED hub activity reports in this period that were pertinent to these topics were collected. The Institutional Review Board (IRB) at the interviewer’s university determined that the interview protocol was not human subjects research since the interviewee was instructed to report on the organization’s perspective, not their personal viewpoint.

### Analysis

The telephone interviews were audio-recorded, transcribed, and anonymized. We used an inductive qualitative analysis approach to identify relevant quotes and themes related to facilitating or impeding TeleED service uptake [14]. The coding process was conducted by a team of three coders at the data coordinating center, including one research associate and two Ph.D. students, who collectively had extensive qualitative analysis and telehealth implementation research experience. Specifically, the research associate has been involved in multiple similar projects with different specialties and organization settings. The first Ph.D. student has assisted in several of these projects, while the second was a new addition to the team with qualitative research experience. The availability of multiple coders with different backgrounds and familiarity with the subject facilitated discussions with valuable perspectives that complemented each other. The team was supervised by the data coordinating center director. The coders read the first two transcripts and independently identified relevant quotes. Coders then met to discuss their findings extensively, which led to a focus on identifying telehealth facilitators and barriers within each of the three implementation stages. We used Microsoft Excel Spreadsheet software to manage and categorize quotes from different hubs into themes and domains.

For this coding activity, facilitators were defined as factors that strengthen service uptake and improve TeleED operations, while barriers are factors that impede usage and create hurdles in TeleED operations. We categorized facilitators and barriers based on the stages of telehealth implementation: The three stages identified in this study are: 1) start-up, which is defined as a phase when telehealth hubs prepare various components that will support their telehealth operations; 2) utilization, which is defined as a phase when telehealth hubs deliver their telehealth services to the spokes; and 3) sustainment, which is defined as a phase when telehealth hubs identify efforts to improve and maintain their telehealth services.

The findings from the first two interviews formed the basis for a coding template. To continue the coding process, all three coders reviewed transcripts from each TeleED hub, one hub at a time, and identified relevant quotes and themes. After each review step, the coders met with the project director to discuss similarities and differences among coders in all identified quotes and themes. Any discrepancies were discussed until consensus was reached. Iteratively, quotes and emerging themes were added to the coding template after each coding step.

After the coding process was complete, the coding team identified domains that categorized themes based on their content similarities. The team performed continuous analysis and review of the data to verify and refine the findings until agreement was reached. We report this study in accordance with the Consolidated Criteria for Reporting Qualitative Research (COREQ) [15].

## Results

The inductive analysis generated 267 unique quotes representing facilitators (66%) or barriers (34%) for telehealth service delivery. Overall, 14% of the quotes represented the start-up stage, 63% represented the utilization stage, and 23% represented the sustainment stage. Across the three stages of telehealth operations, eight domains emerged as the primary components to explain healthcare system operations’ variation. Table 1 lists the themes that were grouped within each domain.

**Table 1:** Main themes and domains for facilitators and barriers within each stage

Domains	Themes	
	**Facilitators**	**Barriers**
	**Start-up Stage**	
**Strategies**	1. Availability of needs assessment 2. Intensive training	1. Incomplete needs assessment 2. Training challenges 3. Systems and technology incompatibility
**Capability**	1. Robust service capacity 2. Experience and Expertise	1. Limited service capacity
**Relationships**	1. Existing relationship	1. Lack of close relationship 2. Referral patterns limitation
**Environment**		1. Competition 2. Politics and contextual issues
	**Utilization Stage**	
**Strategies**	1. Intensive training 2. System and technology enhancement 3. Champions’ availability 4. Adequate staffing 5. Marketing plan 6. Adaptation of TeleED workflow	1. Training challenges 2. System and technology incompatibility 3. Champions’ absence 4. Staffing turnover
**Capability**	1. Robust service capacity 2. Experience and Expertise	1. Limited service capacity 2. Lack of utilization
**Protocols**	1. Well-defined workflow	1. Workflow flaws 2. Referral process limitation
**Service Characteristics**	1. Responsiveness 2. Care coordination 3. Assist with transfer 4. Meeting the needs 5. Video call benefits	1. Time-consuming process
**Relationships**	1. Close relationship 2. Enhancing existing relationship	1. Lack of buy-in 2. Locums not being on board
**Financials**	1. Contracting 2. Grant funding 3. Reimbursements	
**Environment**		1. Lack of integration 2. Culture of transfer 3. Lack of awareness
	**Sustainment Stage**	
**Strategies**	1. Fit with needed service 2. Opportunities 3. Plan in place	1. Poor fit with needed service
**Capability**	1. Availability of multi-specialties 2. Availability of multiple services	1. Lack of multi-specialties 2. Lack of utilization 3. Lack of full-time ED physician 4. Lack of placement options 5. Poor attrition
**Financials**	1. Payment model 2. Contracting 3. Low cost of equipment	1. Payment model flaws 2. Reliance on grant funding 3. Not a money-maker
**Accountability**	1. Evaluation and quality improvement 2. Learning from the experience	1. Lack of evaluation measures
**Relationships**	1. Referral patterns 2. Network resources	
**Environment**		1. Interfering politics and contextual issues

Specifically, the eight domains are defined as the following: 1) strategies—the hubs’ plan of action and efforts related to effective telehealth operations and general hurdles; 2) capability—the hubs’ ability and inability to deliver telehealth services; 3) relationships—the state of connection between hubs and spokes, and how it influences telehealth operations; 4) financials—factors relating to the financial situation affecting telehealth implementation; 5) protocols—aspects related to formal procedure and workflow of telehealth service delivery; 6) environment—the local settings or conditions affecting telehealth operations, such as policies, existing culture, and the availability of other providers; 7) service characteristics—perceived features and quality belonging to the telehealth service; and 8) accountability—factors justifying quality and improvement efforts.

Out of these eight domains, our analysis showed four domains played major roles in influencing implementation at all stages. These domains are strategies, capability, relationships, and environment. The interviewees highlight four primary strategies through multiple implementation stages: conducting a needs assessment, providing training, ensuring technology compatibility with local resources, and fitting the service with local conditions. There are two dimensions of local capabilities that need to be considered when implementing a telehealth service: the health service capacity of local facilities and prior experience in using telehealth. Our findings also showed three primary themes of relationships to consider: the nature of the existing relationship between the TeleED hubs and the spokes, the efforts to strengthen the relationship between facilities, and ensuring buy-in from the local providers to utilize telehealth service. The environment also plays an essential role throughout the implementation stages; our interviewees highlight two themes: the existing policy and local context and existing culture in transferring challenging patients.

**Fig. 1:**
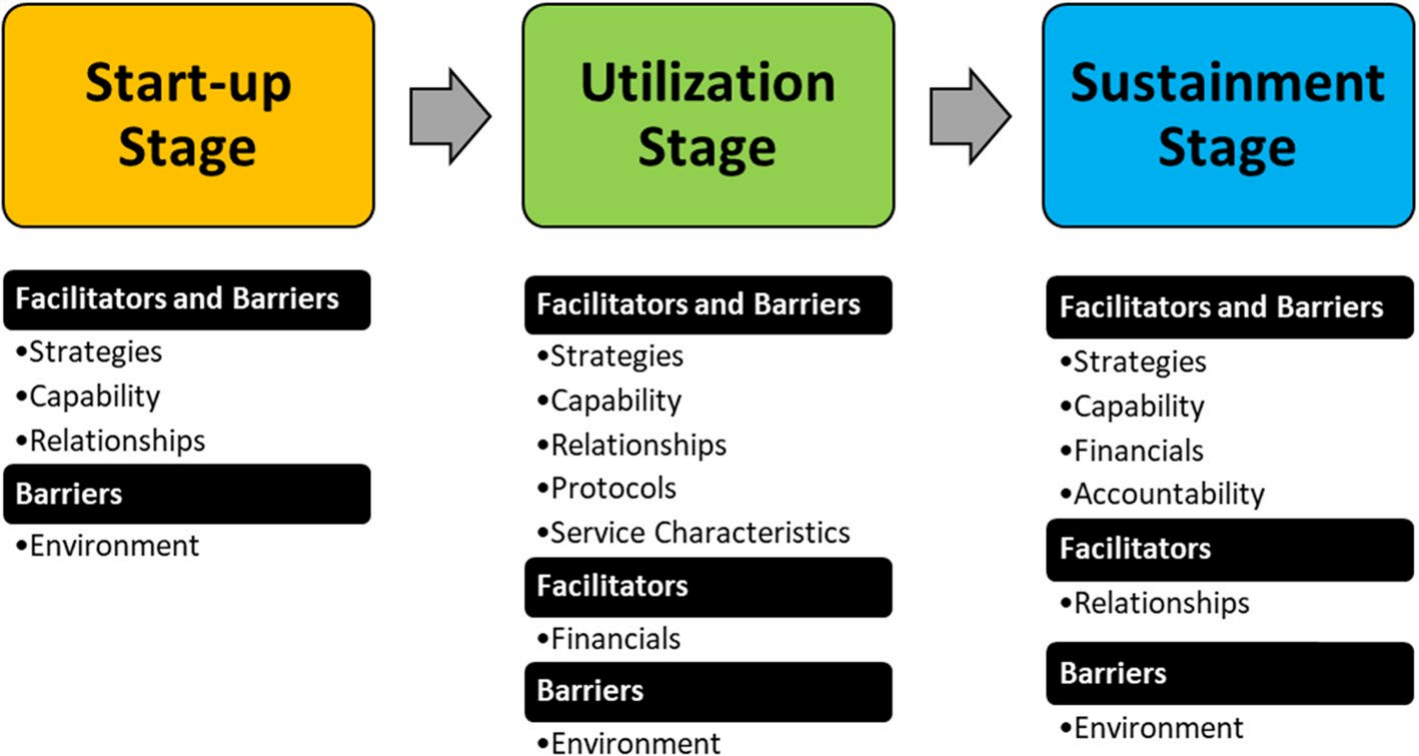
Domains and stages

Furthermore, the analysis indicates that certain domains are aligned with each stage and that not all domains are active in each stage (Fig. 1). The analysis shows that only strategies, capability, and relationships domains in the start-up stage serve as facilitators and barriers, while the environment domain (competition and contextual issues) is perceived only as a barrier. Indeed, the interviewees mention the importance of understanding community needs, ensuring hubs and spokes’ competencies, and forging strong relationships between them to create a strong foundation for telehealth operations. One hub organized a meeting with the local board of health and providers to establish relationships and understand their service area needs. This meeting generated insights on the spoke’s expectations of the TeleED service and the anticipated issues during the implementation process. In contrast, another hub shared the consequences of not conducting a thorough needs assessment and fostering collaboration, which resulted in suboptimal TeleED utilization. Moreover, local policy that limits the scope-of-work for the hub specialists and exclusive alignment of spokes with specific networks hindered the efforts to establish the TeleED service.

Additional domains influence the utilization stage. Strategies, capability, relationships, protocols, and service characteristics domains were identified as facilitators and barriers in this stage. The financial domain exists most often as a part of facilitators, while the environment domain serves predominantly as a barrier. These findings show that as the telehealth implementation stage progressed, more factors influence the TeleED operations. Interviewees highlighted the merit of clear workflow and perceived values of TeleED service, such as motivating care coordination and responsiveness, in facilitating telehealth use in this stage. Interviewees also mentioned that limited-service capacity and lack of buy-in impeded the utilization of telehealth services. The availability of appropriate medical specialty experts and material components are essential to promote utilization.

In the sustainment stage, strategies, capability, financials, and accountability domains serve as facilitators and barriers. The relationships domain is only seen as a facilitator, compared to other stages where it is seen as both a facilitator and barrier. In contrast, the environment domain is consistently perceived as a barrier in the sustainment stage, similar to other stages. Indeed, interviewees consistently mentioned politics and contextual issues as hurdles that demonstrate the challenging climate in implementing telehealth in these settings. Furthermore, our interviewees mentioned well-recognized financial barriers, including a need for a payment model that incentivizes telehealth use, and that rural providers will require a sustainable cost structure that accommodates the low patient volume and initial investment to support the service. Table 2 displays exemplary quotes for themes in each stage to better illustrate the findings.

**Table 2:** Exemplary quotes for facilitators and barriers within each stage

Domains	Themes	
	**Facilitators**	**Barriers**
	**Start-up Stage**	
**Strategies**	**Availability of needs assessment:** “We completed a needs assessment among the rural telehealth network senior leaders. They indicated that the integration of a telemedicine solution to help solve the multifaceted behavioral health issues they were facing was their number one priority.” (Hub 4)	**Incomplete needs assessment:** “We probably should have done a much more detailed needs assessment of the providers in the beginning and built a system that is based on their needs. But we weren’t able to do that.” (Hub 3)
**Capability**	**Experience and expertise:** “The hub specialists have been board certified not only in neurology, but also in stroke neurology. They would be the most qualified to take care of telehealth consults.” (Hub 6)	**Limited service capacity:** “Capacity issues also created a challenge for adding a new telehealth site. There is a lack of human resources to provide services outside of immediate service region due to staff attrition and ongoing position vacancies.” (Hub 4)
**Relationships**	**Existing relationship:** “Each of our existing partners own their fair share of the network and get to decide what goes into it. They consider our telehealth service as part of their main business. So, anything that goes to supporting the service, they will facilitate them.” (Hub 4)	**Referral patterns limitation:** “I think that is where we mis stepped at the beginning. We were not really considering our connections, the existing referral patterns when we built this service.” (Hub 2)
**Environment**	**NA**	**Politics and contextual issues: “**We’ve tried bringing in other specialists, but we face one of the regulatory challenges: they have to be credentialed and privileged. The new specialists are not interested to do that just on a casual basis.” (Hub 1)
	**Utilization Stage**	
**Strategies**	**Intensive training:** “We’ve identified a couple of different instances where those rural sites could use a bit more education. We have also identified that video tutorials would be the most useful for them so we’re planning on doing some short educational videos for these rural EDs to use.” (Hub 2)	**Training challenges:** “During this past year we identified that a weakness of our service was a higher rate of failed telemedicine consults due to lack of training of the clinical night staff, especially locums, at our partner hospitals.” (Hub 5)
**Capability**	**Robust service capacity:** “We have a backup in our telehealth system. And then we even have a backup to the backup, just in case. Because anything can happen. So, we can have two telehealth consults at once. That’s part of being a comprehensive structure. We have to show them that we have that capability.” (Hub 6)	**Lack of utilization:** “We haven’t seen the uptick in referrals as much as we would have liked through our telehealth system because unfortunately a lot of our rural hospital partners are low volume, so they don’t call as much.” (Hub 3)
**Relationships**	**Close relationship:** “Keeping our relationship strong with the physicians, nurses, and administration, and remaining flexible to accommodate the needs of our partner sites has been invaluable throughout this past year and will continue to serve us well throughout the next project year.” (Hub 5)	**Lack of buy-in:** “One of the things that I think we underestimated was how so many of the providers would feel this service was maybe questioning their skills or giving them the sense that we want to be the big brother. Then choose not to use it.” (Hub 2)
**Protocols**	**Well-defined workflow:** “Whenever a hospital here calls about a sick patient, they’ll be connected over telephone with our hub physicians. They’ll briefly talk about the case and then they’ll decide if telemedicine should be initiated. And if so, our physicians will call into their ED, and the unit will automatically answer.” (Hub 5)	**Workflow flaws:** “Our current system isn’t necessarily set up, for that (TeleED) workflow. I mean, it’s an emergency and it could come anytime 24/7/365 so you’ve got to have enough capacity for that, and our workflow is usually 8 am to 6 pm in terms of clinics.” (Hub 6)
**Service Characteristics**	**Care coordination:** “We give them guidance, if somebody’s clearly psychotic or actively suicidal, we say this person is not safe to go home and needs to be admitted. Like I said, the rural providers just want the help, and they are so appreciative of the telehealth service. They are so thankful that we’re there to provide consults.” (Hub 1)	**Time consuming process:** “The providers were resistant because they know it can be fairly time intensive. It’s easy to pick up the phone and just do a phone consultation. They could be driving and have a two or three-minute conversation about a patient. But the telehealth service is a more time-intensive process on the providers.” (Hub 3)
**Environment**	**NA**	**Lack of integration:** “There is limited ability to create a seamless, electronic method for requesting, registering, and completing documentation for the TeleED service visit. All sites largely operated on disparate electronic health record systems. This situation can create a barrier for the adoption and endorsement of telehealth programs.” (Hub 4)
	**Sustainment Stage**	
**Strategies**	**Opportunities:** “It has opened up a lot of other opportunities. With many of these sites we’re able to do other telemedicine things, services with them, so it was kind of a side benefit in that it was just opening up the conversation about telemedicine. There have been a lot of little side conversations that I think have been very beneficial for all of these sites.” (Hub 2)	**Poor fit with needed service:** “The only way to extend this long term is that we would have to have additional providers available that fit with rural hospitals’ needs.” (Hub 3)
**Capability**	**Availability of multi-specialties:** “We do tele-stroke and tele-burns as well. And we have other ancillary services such as our language line and sign language interpretation. We are planning to keep adding more value to our telehealth service.” (Hub 1)	**Attrition:** “We found attrition of providers in community health centers at some of our sites, they just don’t have enough people to be able to cover what the needs are.” (Hub 4)
**Relationships**	**Referral patterns:** “We have a lot of referrals from sites that currently are not in our TeleED partners list. Those are potential sites for the telehealth service.” (Hub 6)	**NA**
**Financials**	**Payment model:** “That’s one of the big things that has set us apart (only charging for the professional fees). We were looking at something that really doesn’t cost the care site anything.” (Hub 2)	**Not a money maker:** “Right now the continuation contract went back and forth, and it’s in spokes’ hands. They can’t complain about the price because we are practically giving the service for free. If there’s still a concern about it, I’m not so sure we’d be able to sustain the service.” (Hub 6)
**Accountability**	**Evaluation and quality improvement:** “We have started to collect information on why physicians decide to deviate from using the telemedicine service according to the protocol to understand the important clinical reasons driving these decisions, the appropriateness of when to activate telemedicine, and improving the use of telemedicine services.” (Hub 5)	**Lack of evaluation measures:** “The providers that are involved are not as invested in the hospital. The administrators do not have measures about transfers or about patient centeredness -as far as could you avoid the transfer or could we have found a different option for this patient- which are important for improvement purposes.” (Hub 3)
**Environment**	**NA**	**Politics and contextual issues:** “We’ve heard from our legal staff that there’s some special blessing with grants, but you know they go away. So, that’s part of the fear of how you make something that’ll be sustainable because a lot of specialists are a little daunted by the amount of hurdles and paperwork they’ve got to do to provide the service.” (Hub 1)

## Discussion

The implementation of healthcare innovation is not always successful and can be influenced by multiple factors. The experience of six U.S. healthcare systems in implementing TeleED across 65 rural hospital emergency departments (EDs) over three years shows different aspects that have promoted or impeded telehealth adoption and utilization. We found eight domains (strategies, capability, relationships, financials, environment, protocols, service characteristics, and accountability) that can act as facilitators and/or barriers for telehealth operations in rural EDs. Moreover, we identified three stages of telehealth operations (start-up, utilization, and sustainment) and highlighted specific themes that operate within each domain. Our findings show that even when the implementation of TeleED across healthcare systems are going through similar stages, local factors influence the process. We expect by appreciating and responding to the eight domains, healthcare systems can achieve more predictable and favorable telehealth implementation outcomes in rural EDs.

In the start-up stage, our respondents highlighted the importance of completing a needs assessment and intensive training as initial strategies to prepare facilities for telehealth services. Indeed, the finding is consistent with evidence from other studies, which show that identifying local health priorities followed with sufficient education can be useful to facilitate telehealth service development and implementation [16–18]. However, the lack of supportive policies and frameworks for virtual and collaborative consultations created a challenge in realizing the benefits of telehealth; identifying these potential barriers in the start-up stage may guide preemptive steps to minimize their impact on implementation [19–21]. Moreover, differences in healthcare systems’ affiliation among rural hospitals can potentially be addressed by establishing cross-system collaboration to facilitate telehealth in rural areas that need the service. This strategy is important because rural hospitals frequently operate with less resources compared to their urban counterparts. Literature has highlighted collaboration and partnerships as a key to bridge the healthcare gap in rural areas [22–24].

The utilization stage is bolstered by the availability of well-defined workflows and telehealth service characteristics such as facilitating care coordination, improving patient care responsiveness, assisting with transfers, meeting the medical needs, and video call benefits. Indeed, access to proper guidelines and creating clear workflows that eliminate multiple interpretations are essential factors influencing innovation utilization [25]. Simultaneously, the local administrators’ and clinicians’ engagement is crucial since their buy-in may overcome lack of use, technology issues, negative perceptions, and lack of resourcing [21, 26]. Some of the barriers in this stage are the lack of system integration, existing transfer culture, and limited awareness. These barriers are frequently mentioned and encapsulated within the environment domain perceived as barriers across stages (i.e., start-up, utilization, and sustainment). Studies have shown that these barriers might be driven by the healthcare facilities’ inclination towards preserving the existing care delivery model with known risks, routines, and regulations to maintain stability, which are usually absent with the adoption of new technology or innovation [27, 28].

In the last stage, sustainment of service, financials, and accountability-related themes were prominent and influential. Adopters’ success in technology dissemination is influenced by identifying the new practice's cost and benefit for the local providers and whether there is support for rural financial constraints [25, 29]. These learning points are important for implementation in rural EDs because sustainability of an innovative program has been a persistent challenge in rural areas [30]. This issue stems from neglecting the rural context in the implementation process, inconsistent funding, and disjointed monitoring. To address these challenges, continuous evaluation and quality improvement as well as learning from experience can positively influence sustainability [31].

The use of inductive analytical approach in this study has identified novel insights that were organically generated from the experience of implementing TeleED in rural EDs. Four strategies were performed to establish rigor in this qualitative study [32]. First, we developed a coding system that outlined interviewees’ quotes into themes, domains, and stages. Second, we used multiple coders to analyze and discuss the transcripts to ensure the findings are a synthesis of multiple viewpoints. Third, we acknowledged and elaborated on the coders’ background and experience to address concerns of researcher bias. Fourth, we identified negative cases and included them in our analysis and discussions to provide a comprehensive perspective on the implementation of TeleED in rural hospitals.

This study had several limitations. First, the interviews were limited to the hub sites. Additional insights on the implementation process from the spoke sites may emerge if they were interviewed on this topic. Second, the hub sites had differences in their organization characteristics and specialties. A similar type of organization may generate additional findings and specific recommendations that cannot be explored and provided in this study. Third, an inductive qualitative analysis approach was used to explore emerging themes instead of using an existing implementation model to frame a deductive qualitative analyses approach. Using a deductive approach would have permitted further testing of existing models to explore how those models performed in terms of this clinical telehealth application.

## Conclusions

Overall, our study contributes to the field by highlighting factors that influence telehealth operations in rural EDs. This study outlines implementation strategies needed to manage innovation in an organization. Understanding the facilitators and barriers of TeleED operation may help healthcare leaders successfully adopt and disperse innovation across stages of implementation. Moreover, this study’s findings may inform policymakers in creating policies that motivate telehealth innovation diffusion.

## Data Availability

The datasets used and/or analyzed during the current study are available from the corresponding author on reasonable request due to privacy of our interviewees.
